# Supplementary material for: Assessment of the reliability, responsiveness, and meaningfulness of the scale for the assessment and rating of ataxia (SARA) for lysosomal storage disorders
Source: J Neurol. 2024 Sep 3;271(10):6888–902. doi: 10.1007/s00415-024-12664-y (PMC11447074; doi:10.1007/s00415-024-12664-y)
Supplement: Supplementary file 1 — Supplementary file1 (DOCX 15 KB) [file 415_2024_12664_MOESM1_ESM.docx]

**Supplemental Material I – Exit Interview Template**

**Phase I**

1. Please describe in your own words your (or the patient’s) NPC symptoms before the IB1001-301 trial:
2. Please describe in your own words how your (or the patient’s) NPC symptoms affected your* everyday life before the IB1001-301?

**Phase II**

1. Please describe in your own words how your (or the patient’s) experience with NPC symptoms changed during the IB1001-301 clinical trial?
2. Please describe in your own words any improvements (of any magnitude) you observed during the IB1001-301 clinical trial?
3. (If applicable) Please describe in your own words how these improvements (of any magnitude) were relevant/meaningful to you/ your* everyday life?

**Phase III**

1. Please describe in your own words any differences (of any magnitude) observed in in your (or the patient’s) NPC symptoms during Period I versus Period II?
2. (If applicable) Please describe in your own words how these differences (of any magnitude) were relevant/meaningful to you/ your* everyday life?

**If completed by the caregiver, please describe how these symptoms affected both the caregiver and patient’s everyday life.*
